# Processing time not modality dominates shift costs in the modality-shifting effect

**DOI:** 10.1007/s00426-019-01276-1

**Published:** 2019-12-14

**Authors:** Hettie Roebuck, Kun Guo, Patrick Bourke

**Affiliations:** 1 School of Psychology, University of Lincoln, Lincoln, LN6 7TS UK; 2 University of Wisconsin, Madison, 53706 USA

## Abstract

Shifting attention between visual and auditory targets is associated with reaction time costs, known as the modality-shifting effect. The type of modality shifted from, e.g., auditory or visual is suggested to have an effect on the degree of cost. Studies report greater costs shifting from visual stimuli, yet notably used visual stimuli that are also identified slower than the auditory. It is not clear whether the cost is specific to modality effects, or with identification speed independent of modality. Here, to interpret whether the effects are due to modality or identification time, switch costs are instead compared with auditory stimuli that are identified slower than the visual (inverse of tested previously). A second condition used the same auditory stimuli at a low intensity, allowing comparison of semantically identical stimuli that are even slower to process. The current findings contradicted suggestions of a general difficulty in shifting from visual stimuli (as previously reported), and instead suggest that cost is reduced when targets are preceded by a more rapidly processed stimulus. ‘Modality-Shifting’ as it is often termed induces shifting costs, but the costs are not because of a change of modality per se, but because of a change in identification speed, where the degree of cost is dependent on the processing time of the surrounding stimuli.

## Introduction

Having to switch attention between visual and auditory targets is associated with switching costs, known as the modality-shifting effect. When a previous target is of a different modality detecting a subsequent target is slower than when it is of the same modality (e.g., Zubin, 1975; Ferstl, Hanewinkel, and Krag, 1994; Spence, Nicholls, and Driver, 2001; Lukas, Phillip, and Koch, 2010). The frequency and recency of a given modality are thought to build up an underlying prediction of what the next target will be (Epstein & Rock, 1960). The Configuration–Execution model suggests that top–down factors (e.g., expectancy) affect the time to program central mental operations, and bottom–up factors (e.g., recency) independently affect the time to execute them (Ruthruff, Remington, & Johnston, 2001). Shifting costs represent the delay when a switch in modality conflicts with the underlying prediction generated from the set of previous stimuli.

The size of the shift cost is not always the same. It is suggested that the type of modality that is being shifted from can have an effect on the degree of cost induced onto the subsequent target. For example, it has been suggested that there is either an underlying visual dominance, or a difficulty to shift away from visual stimuli compared to auditory stimuli (Posner et al., 1976). Visual stimuli are suggested to be not as attention capturing as auditory (e.g., Cohen & Rist, 1992); thus, visual dominance may result in a greater attentional tuning to the visual modality (Posner, Nissen, Klein, 1976). In the context of modality-shifting, a selective bias towards visual stimuli is thought to benefit responses to repeated trials, resulting in a greater cost if the modality switches (e.g., Miller & Cohen, 2001; Lukas et al., 2010). The distinction between the possibility of an underlying visual dominance or a difficulty to shift away from visual stimuli has been explored further comparing modality-shifting across multiple modalities (Spence, Nicholls, & Driver, 2001). Using tactile (finger stimulation), visual (light illumination) and auditory stimuli (pure tones), participants had to make a left/right discrimination for each modality. When the modality of discrimination shifted to another modality, there were even greater reaction time (RT) costs associated with shifting away from touch, compared to both visual and auditory stimuli. From these findings, they conclude that it is even more difficult to shift attention away from the tactile modality, rather than an underlying predisposition to attend to visual. Therefore, the authors interpret the findings observed by Posner et al. (1976) as a greater difficulty to shift away from visual stimuli, rather than an overall visual dominance, as tactile stimuli could induce even greater shifting delays compared to the visual stimuli.

The debate over whether there are difficulties to shift away from a modality, or whether a modality dominates has also attracted interest in the interpretation of underlying difficulties associated with different clinical disorders. Both in terms of deficiencies, which may be specific to a modality (e.g., in Huntingtons Disease; Sprengelmeyer, Lange, & Hömberg, 1995), and sensory dominance to a modality (e.g., in Schizophrenia; Zahn, Pickart, & Haier, 1994).

Despite the interest in modality specific effects, there has been little consideration to what extent switching costs are specifically inherent to modality verses a switch associated with the processing requirements of a differing stimulus. Studies suggest that task demands can influence the degree of shift cost, for example, if a task requires temporal processing or spatial processing, responses can be affected depending on which modality is given priority (Lukas, Philipp, & Koch, 2014). It is possible that differences between modality in attention shifting tasks may have less to do with whether they are auditory, visual or tactile and more to do with how quickly they can be processed. The impact of speed of stimulus identification has not previously been considered in the context of modality-shifting. However, research suggests that more caution is needed when attributing differences in performance to the modality of presentation more generally (Roebuck, Freigang, & Barry, 2016). The findings show that in a simple GO NOGO continuous performance task, different types of stimuli within a modality can induce different response rates and error propensities, suggesting that effects of stimulus type may be being misinterpreted as modality effects. Specifically, participants were presented with two stimuli, and had to press a button to one stimulus, and inhibit their responses to the other. Slower RT’s and greater errors were observed in the condition using two auditory tone stimuli (high and low tones), compared to the same task (and modality) using auditory spoken sounds (X and O). The stimuli were all presented for the same duration, but the linguistic stimuli may be easier to categorically identify in memory, and faster to represent. Such findings are limited to not only the choice of stimulus, but also the perceptual quality. Low intensity but detectable auditory stimuli take longer to process than the same stimuli presented at a comfortable listening level, and are associated with increased errors of attention and inhibition (Roebuck, Guo, & Bourke, 2015). It appears that one key element in which differences between stimuli may be observed is in the speed of processing, which can depend on the type of stimulus selected, not just the modality.

Visual and auditory variants of the same task are often used to understand the relationship of attention shifting between modalities. The wide range of stimuli used to represent auditory and visual domains are likely to be problematic in making these comparisons because they differ on more dimensions than simply the sensory modality they are presented in Roebuck et al. (2016); Roebuck, Sindberg, & Weismer, (2018). With this in mind, it is possible that the interpretations of the findings from modality-shifting tasks may be affected by the stimuli selected, and the speed at which they can be identified, rather than just the modality itself. Detecting a sound is thought to have an alerting effect and happens more rapidly than detection of a visual cue (e.g., Posner et al., 1976), but when stimuli must be processed for their meaning, RTs are longer than simple detection. For example, lexical semantic processing takes longer with auditory stimuli, related to the serial production of the sound to derive meaning (Holcomb & Neville, 1990). Notably, however, modality-shifting tasks have used stimuli with slower physical detection of the visual stimuli compared to the sound, e.g., detection of white noise compared to light illumination (Spence & Driver, 1997). Slower identification of visual stimuli has been consistently reported in the tasks which also describe greater difficulty to shift from visual stimuli (e.g., Posner et al., 1976; Ferstl et al., 1994; Zahn, Pickart, & Haier, 1994, Sprengelmeyer, Lange, & Hömberg, 1995). Given that the frequency and recency of preceding targets appear to create an underlying expectation about what, and when to expect a subsequent target (Epstein & Rock, 1960), perhaps the greater difficulty to shift away from visual stimuli may be due to the speed in which they are identified. The impact of temporal preparation is suggested in other paradigms whereby participants respond faster to a visual target when it is accompanied by a task-irrelevant auditory alert. It is suggested that the quickly identified auditory alert speeds the response of the visual target (Los & Van der Burg, 2013). The auditory alert (that can be identified more quickly) acts as a warning cue, which allows preparation for the more sluggish visual target to be initiated. By manipulating the delay between the signal and target, and equating the effective preparation period, the authors show the effect to be a consequence of temporal preparation rather than multisensory integration. In modality-shifting paradigms, the switch cost may be related to the slower bottom up timing created by the speed of processing the visual stimuli, rather than inherently due to shifting from visual to auditory. Therefore, to interpret whether modality-shifting is truly about modality, stimuli can be compared whereby the visual stimuli can be identified faster, rather than the auditory. If speed of identification is critical rather than modality, the reverse effect of shift cost would be predicted.

In the current study, a continuous performance task paradigm will be used to allow us to further understand the modality-shifting effect to tease apart modality effects from effects associated with how fast the particular stimulus can be processed. In the Sustained Attention to Response Task (SART), a participant must respond with a button press to frequently presented targets (digits 1–9), and withhold a button press to one rarely presented NOGO target (e.g., 3) (Robertson, Manly, Andrade, Baddeley, & Yiend, 1997). The timing and expectation of a response are thought to be fundamental to why errors are made. The frequent and regular requirement to press a button sets up an internal prediction for a motor response to be made within a highly anticipated timeframe, often leading to a ‘‘false alarm’’ when a rarer unexpected target is presented (Chamberlain & Sahakian, 2007). These ‘impulsive’ errors are thought to be made when the time needed to fully identify the stimulus takes longer than the time allocated to initiate the highly anticipated motor response (Logan & Cowan, 1984). The more established a required response in a frequent and regular pattern, the more anticipated the response at a specific time (Chamberlain & Sahakian, 2007). As such errors may be related to an internal speed–accuracy trade-off (Peebles & Bothell, 2004; Helton, 2009).

In contrast to the stimuli used in modality-shifting tasks, comparisons using a SART paradigm found that the auditory stimuli were responded to more slowly than the visual stimuli, when presented in isolation (Seli, Cheyne, Barton, & Smilek, 2012). Even when the stimuli are semantically the same between the visual and auditory domains (e.g., digits), there are apparent differences in how quickly targets may be identified. With these stimuli, the identification stage may be faster because all the information needed to identify the visual digit is available throughout the presentation, whereas an auditory digit takes time to reveal as a spoken word, and so the identification occurs serially (Shen & Mondor, 2006). Using a SART design would, therefore, allow the utilization of stimuli where the visual stimuli are naturally identified more quickly than the auditory.

**Fig. 1:**
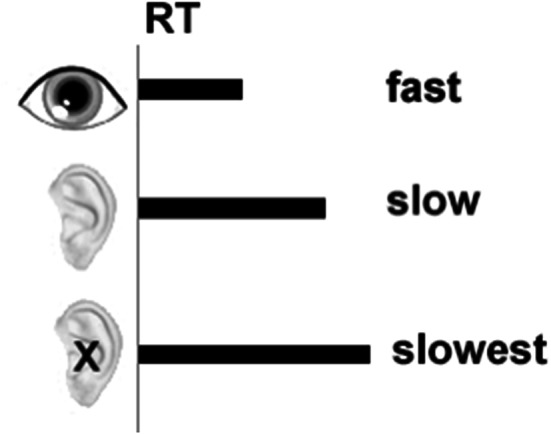
Illustration of RT across stimulus types for the current study. Horizontal lines represent duration of RT from time of stimulus onset depicted by the gray vertical line. Visuals are exaggerated for illustration purposes only; the eye represents the RT for a visual target, the ear an auditory target, and the ear with an X a quiet auditory target

The SART paradigm will be modified to use both visual and auditory target digits requiring ‘modality-shifting’, where the auditory targets are identified slower than the visual. A second condition will use the same visual and auditory targets, but the auditory targets will be presented at a low but detectable volume. Using the same auditory targets in a second condition presented quietly provides a comparison of a stimulus that takes even longer to process (Roebuck et al., 2015), but are otherwise identical, both semantically and in modality to the auditory stimulus presented at a normal intensity (see Fig. 1 for an illustration).

**Fig. 2:**
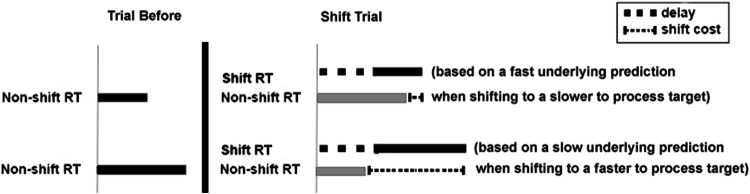
Illustration of shift and non-shift RT. Horizontal solid lines represent duration of RT from time of stimulus onset depicted by the gray vertical line. The large dotted line represents the consistent shift, and the fine dotted line represents the changing shift cost. With this model, smaller shift costs are predicted when the trial before is faster to process

Such a design allows the comparison of (a) modality, where the identification time is faster for the visual stimuli (inverse of tested previously), (b) the cost of shifting to/from stimuli that differ in the speed in which they can be identified. The impact of speed of response expectation can be investigated by comparing responses to targets following a ‘shift’ (a cross-modal trial). If as previously suggested there is an underlying difficulty to switch away from the visual modality (e.g., Posner et al., 1976), we would expect greatest costs to both of the auditory targets (normal volume and low) following the visual targets. Alternatively, if processing time is critical, then we would instead predict the reverse relationship, with smaller costs for the auditory targets (processed slower) following the visual one (processed faster) due to the underlying predicted timing based on the temporal preparation predicted from the previous target (see Fig. 2). The conflict-monitoring hypothesis proposes that the systems that sub-serve cognitive control include an evaluative system, which appraise the current demands and conflicts in processing (Botvinick, Braver, Barch, Carter, & Cohen, 2001). Figure 2 shows the asymmetry in RT that we predict to emerge when switching between a ‘fast to process’ stimulus to a ‘slow to process’ stimulus. In this case, the top line shows a constant delay resulting from a conflict in a change of target, followed by a response based on a fast underlying prediction. The line below shows the non-shift RT of the target. The size of the shift cost is calculated by subtracting the RT of the trial following a stimulus shift from the average RT of that stimulus type from non-shift trials. Here, the size of the shift cost would be predicted to be small (see fine dotted line as an example). In contrast, the second example in Fig. 2 shows a switch from a ‘slow to process’ stimulus to a ‘fast to process’ stimulus. Here, you can see the same reprogramming shift delay as the first example, but this time with a slow underlying prediction created from the previous target. A slower response would be predicted, resulting in a larger shift-cost when calculated as a difference from the normally fast RT of the non-shift target (second example in Fig. 2). Based on this framework, we would predict the smallest costs when switching from the visual targets to the low-intensity auditory targets (processed slowest).

Using a SART paradigm also allows the impact of processing time to be explored further by also having a measure of inhibition errors following a switch. The regularly anticipated responses in a SART frequently result in erroneous responses to NOGO targets when a motor response is routinely initiated before a target is fully processed (Hitchcock & Dember, 1999). In this case, the RT delay caused by a shift in modality may be an advantage, by interrupting the automaticity of engrained responses, and allowing time to fully identify the target. We would, therefore, predict more commission errors following trials of the same modality compared to when there is a change in modality. When NOGO trials occur following a shift, we would predict more errors to occur when there is a switch with a smaller delay compared to a larger delay. If as has been found in previous studies, where smaller delays are observed to visual targets, we would predict highest commission errors to visual targets. If the dominance in modality-shifting is affected by processing time, rather than being specific to a modality, with smaller delays to auditory targets, we would instead predict more commission errors for auditory targets.

## Materials and methods

### Ethics statement

This study was approved by the School of Psychology Research Ethics Committee at the University of Lincoln. Informed consent was obtained from all individual participants included in the study. All experimental procedures complied with the British Psychological Society Code of Ethics and Conduct and with the World Medical Association Helsinki Declaration as revised in October 2008.

### Participants

Thirty participants were recruited for the study. Participants were excluded as outliers if they made errors more than 1.5 of the inter-quartile range in the task. Five participants were excluded on this basis ensuring all participants included could do the task. Inclusion criteria specified no known hearing impairments or attention deficits. In addition, participants were also given a hearing test prior to testing (see “Determining lowest detection thresholds” below). No exclusions were made on these criteria. Twenty-five participants (21 female, 18–44 years, *M* = 21, SD = 6) were included in data analysis.

### Design

**Fig. 3:**
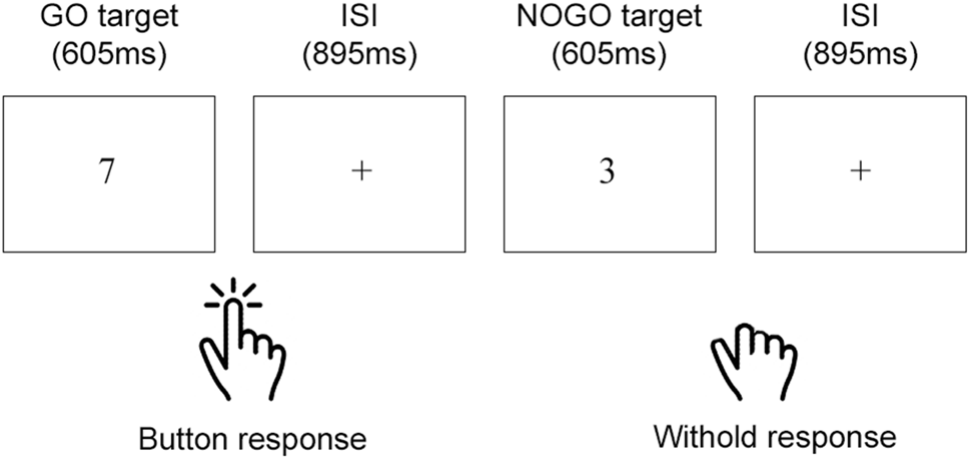
Illustration of two trials in a SART paradigm

Participants undertook a modified version of the SART (Robertson et al., 1997) using visual and auditory targets within the same task rather than only visual targets. In the SART, a participant must press a single button to frequently presented targets (digits 1–9), and withhold their response to a rarely presented NOGO target (e.g., 3). The button is the same regardless of the targets or the modality. See Fig. 3 for an illustration. The experiment used a repeated-measures design with two conditions. One condition used clearly presented visual digits and spoken digits at a ‘normal listening volume’ associated with speech levels (60 dB sound pressure level (SPL), Pearsons, Bennett, & Fidell, 1977). In other words, both the visual and auditory digits were easy to detect. We expect that the visual stimuli will be faster to detect than the auditory stimuli (Seli et al., 2012). Providing a comparison to other tasks that have used stimuli where the visual stimuli are detected more slowly than the auditory (e.g., Posner et al., 1976; Ferstl et al., 1994; Zahn et al., 1994, Sprengelmeyer et al., 1995). The other condition used clearly presented visual digits and spoken digits at ‘lowest detection threshold’ (for each individual participant). In this condition, the visual stimuli were the same as the first condition, but the auditory stimuli were presented quietly (but could be heard). We expect that the auditory stimuli will be detected even more slowly when presented at the quiet intensity. The volume intensity manipulation varied by condition, normal listening volume targets and lowest detection threshold targets were not presented in the same task. All participants completed both conditions but the order was counterbalanced. Participants were presented with a random sequence of auditory and visual digits (i.e., numbers one–nine) presented at regular intervals. Participants were required to withhold response to one specified number (NOGO trial), and initiate a response to all other numbers (GO trials), in both modalities. No feedback was given. The presentation of auditory and visual targets was randomized.

### Stimuli and apparatus

Auditory stimuli were spoken by a non-computer generated female voice with normalized volume presented equally in both ears through headphones. Participants were seated in front of a desk with a 17″ monitor, and a screen resolution of 1024 × 768 pixels. At a viewing distance of 57 cm, the monitor subtended a visual angle of 29° × 35°. Visual stimuli were presented centrally in bold black Times New Roman font, 200-point font size, on a white background. Equal proportions of auditory and visual stimuli were presented; 261 auditory stimuli, and 261 visual stimuli, within each condition. Each number was presented randomly and lasted for 605 ms with an inter-stimulus interval (ISI) of 895 ms. Stimuli were presented independently (auditory and visual stimuli were never presented at the same time). Participants were able to respond any time within the presentation of the stimulus and the ISI (total time 1500 ms). Presentation time of stimuli was the same in both modalities. Each of the two conditions lasted for 13 min and consisted of 522 trials. This included 58 NOGO targets, corresponding to one specified number, e.g., 3. This was made up of 29 auditory NOGOs and 29 visual NOGOs. Participants were asked to inhibit their response for this number. The NOGO number, e.g., 3 was fixed across the two volume conditions for each participant, but the NOGO number was randomized between participants. There were 464 specified ‘GO’ targets which were the eight remaining number stimuli, e.g., 1, 2, 4, 5, 6, 7, 8, 9, requiring participants to press the response button. 232 were auditory and 232 were visual. During the experiment, a black fixation cross was presented in the center of the screen during ISIs and whenever a visual stimulus was not present to maintain the gaze of the participants to be task focused.

### Procedure

At the beginning of the task, a start screen was presented, with a reminder of the correct key to press. Participants were instructed to rest their finger ready on the single response key. The participant initiated the start of the experiment by pressing the key themselves to ensure that they were ready to begin. After 2 s, the targets were presented in a random sequence, with the auditory and visual presentation of stimuli also randomized. Participants then undertook the second condition. The order of tasks was counterbalanced between participants.

### Determining lowest detection thresholds

Prior to experimentation, all participants had a standard hearing test using an Oscilla SM930 screening memory audiometer. A single handheld thumb press response button was used. Participants were seated in a sound proof booth and tested independently. Tones were presented through passive noise reducing headphones (TDH39 headphones SILENTA noise reducing headset). The same closed-cup headphones were also used for the experiment. To be included in the study, all participants were required to have normal bilateral pure tone hearing thresholds of 25 dB HL or better for frequencies: 250, 500, 1000, 2000 and 4000 Hz. The ambient sound levels within the booth complied with the specifications according to British Standards/European Norm (BS EN) ISO 8253-1:1998 which allow thresholds as low as 0 dB HL to be established. In addition to ruling out any unknown hearing difficulty, the audiogram provided a starting point to specify the level for the low-intensity condition. To establish the volume of stimuli for the low-level condition, participants were played a sequence of the stimuli numbers from one to nine in a random order. If participants were unable to repeat all of the digits, the intensity of the stimuli was increased by 5 dB SPL. If participants accurately identified all digits, the level was reduced by 5 dB SPL and another sequence of digits played. The level was reduced until participants were no longer able to identify all of the numbers. The intensity 5 dB SPL above was then tested again. The low volume condition was defined as the lowest level at which the participant could accurately identify 100% of the numbers. The level was checked twice with 100% accuracy required on both occasions as per the procedure in Roebuck et al. (2015).

### Analysis

All analysis on RT was conducted on correct responses. Analyses were conducted on stimulus modality (visual and auditory targets), volume intensity (normal and low) and transition type (shift and non-shift trials). Shift trials were trials where the target was a different modality to the target before. Non-shift trials were trials where the target was the same modality as the target before. To interpret the delay following a change in stimulus, the shift cost was calculated by subtracting the RT of the trial following a stimulus shift from the average RT of that stimulus type from non-shift trials. Analysis on commission errors (false alarms) was also conducted using the same IVs. Repeated-measures ANOVAs were conducted in SPSS. Post hoc tests were run with Bonferroni adjustments for multiple comparisons.

## Results

### Role of stimulus type and speed of response to correct GO targets

To interpret the effect of processing time, a repeated-measures ANOVA compared RT to correct GO targets between transition type (shift and non-shift), volume condition (normal and low) and modality of the previous target (auditory and visual). There was a significant main effect of transition type *F* (1, 24) = 121.82, *p *<.001, *η*_p_^2^ = .84. Consistent with previous findings, RT was slower after a shift trial (*M* = 659, SD= 86 ms) compared to a non-shift trial (*M *=587, SD= 79 ms). There was also a main effect in RT for the factor volume *F* (1, 24) = 5.16, *p *=.032, *η*_p_^2^ = .18. In line with prediction, responses to targets presented in the low condition were slower (*M *= 633, SD= 77 ms) than those in the normal volume condition (*M *= 613, SD= 88 ms). There was a main effect of the modality of the previous target *F* (1, 24) = 60.04, *p *< .001, *η*_p_^2^ = .71, contrary to previous studies, responses were slower following an auditory target (*M *= 641, SD= 89 ms) than after visual targets (*M *= 604, SD= 76 ms).

**Fig. 4:**
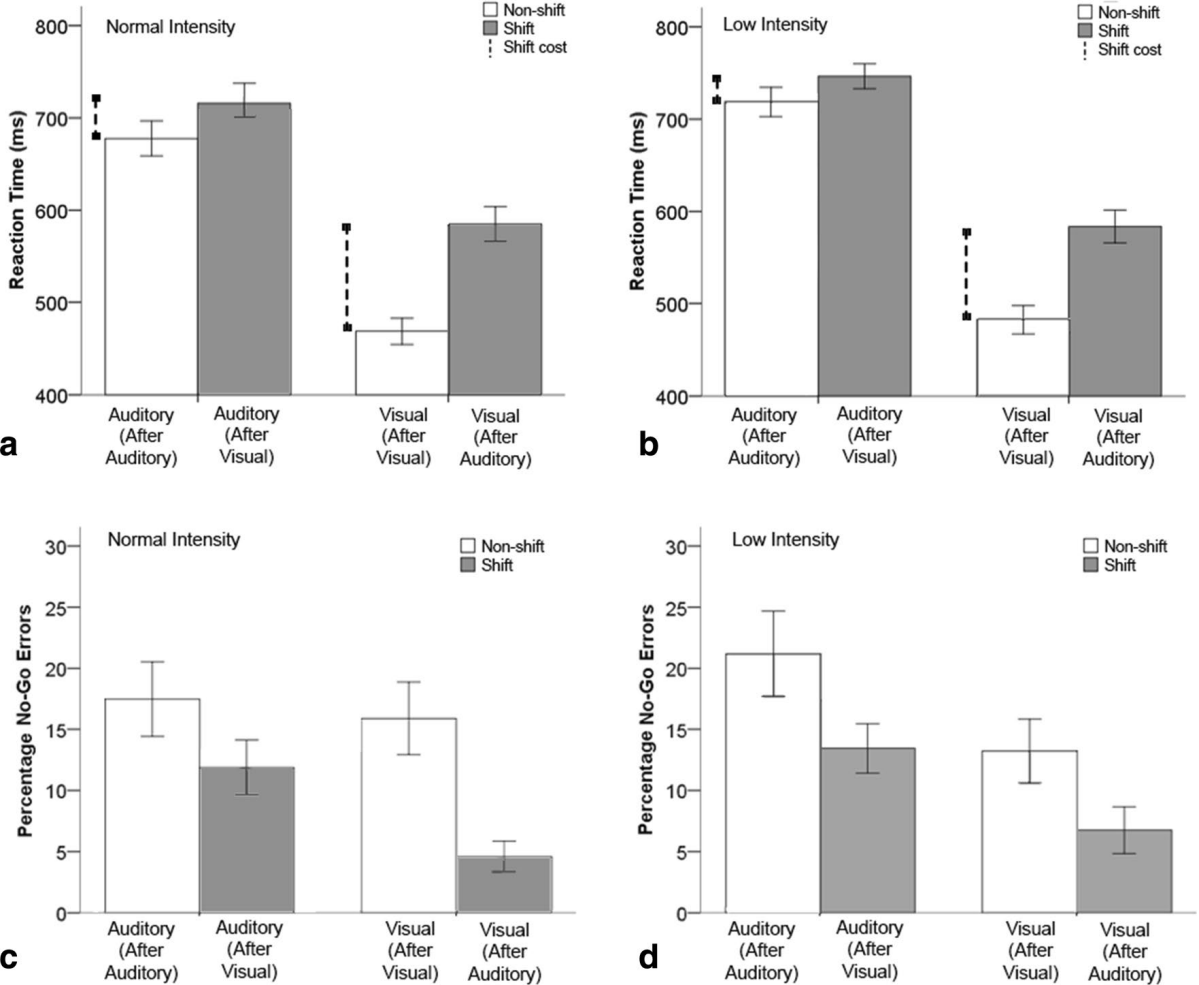
**a** RT to auditory and visual digits in the normal volume conditions to shift and non-shift GO trials. **b** RT to auditory and visual digits in the low volume conditions to shift and non-shift GO trials. Error bars are ± 1 SE. Shift cost is marked in vertical capped dashed lines. **c** Percentage shift and non-shift NOGO errors to auditory and visual digits in the normal volume condition. **d** Percentage shift and non-shift NOGO errors to auditory and visual digits in the low condition. Error bars are ± 1 SE

There was also an interaction between the modality of the previous target and the transition type *F* (1, 24) = 449.19 *p *< .001, *η*_p_^2^ = .95. For non-shift trials RT was slower for auditory–auditory targets (*M * = 698, SD = 86 ms) compared to visual–visual (*M *= 476, SD= 73 ms) (*p *< .001, Fig. 4a and b). For shift trials (Fig. 4a, b), RT was slower for visual–auditory (*M * = 733, SD = 79 ms) compared to auditory–visual (*M *= 584, SD= 92 ms) (*p* < .001, Fig. 4b).

There was also an interaction effect between transition type and volume *F* (1, 24) = 7.87 *p *=.01, *η*_p_^2^ = .25. For non-shift trials, RT was slower in the low intensity condition (*M *= 601, SD = 76 ms) than non-shift trials in the normal intensity condition (*M* = 573, SD = 83 ms) (*p * = .006). For shift trials, there was no significant difference if the volume intensity was low (*M *= 665, SD = 79 ms) or normal intensity (*M *= 652, SD = 92 ms) (*p* = .19).

There was a three-way interaction between modality of the previous target, transition type and volume condition *F* (1, 24) = 8.96 *p * = .006, *η*_p_^2^ = .27. As we would expect, the significant difference in non-shift reaction time between conditions (normal intensity and quiet) is driven by the difference in auditory stimuli (normal intensity (*M * = 678, SD = 95 ms) vs low intensity (*M  *= 719, SD = 76 ms) (*p* = .003) which differ between conditions. The speed of response to non-shift visual stimuli does not significantly differ when they occur within a condition of differing auditory stimuli [normal intensity (*M  *= 469, SD = 71 ms) or low intensity (*M  *= 483, SD = 75 ms) (*p* = .14)]. When a shift occurred to a visual–auditory, RT was slower in the low intensity condition (*M * = 747, SD = 68 ms) compared to a visual–auditory target in the normal intensity condition (*M *= 719, SD = 90 ms) (*p * = .035). When a shift occurred to an auditory–visual target, RT did not differ between the quiet intensity (*M  *= 584, SD = 89 ms) or the normal intensity conditions (*M * = 585, SD = 95 ms) (*p* = .88).

### Role of stimulus type on RT shift cost

Shift costs are the difference between the non-shift RT for that target, and the RT of the target that has been shifted to. Shift costs were compared between stimulus type (visual–auditory and auditory–visual) and volume condition (normal and low). There was a significant main effect of stimulus type *F* (1, 24) = 60.04, *p *< .001, *η*_p_^2^ = .71. The direction contrasts with previous findings, but supports our prediction for processing time rather than modality; shift costs were greater for auditory–visual digits (*M* = 109, SD = 39 ms) than visual–auditory ones (*M* = 34, SD = 49 ms) (*p *= < .001, Fig. 4). There was also a main effect in RT for the factor volume *F* (1, 24) = 7.87, *p* = .010, *η*_p_^2^ = .25. Shift costs in the normal volume condition were greater (*M* = 79, SD = 56 ms) than those in the low condition (*M* = 64, SD = 60 ms) (*p* = .01).

### Relationship between differences in stimulus identification time and degree of cost

Further analysis assessed whether the difference in RT between the auditory and visual targets was related to the degree of cost. By subtracting each participants’ average auditory non-shift RT from their visual non-shift RT, we had a difference score indicating how much slower they were at identifying auditory targets. This method tests differences in RT between modalities independent of their average RT. A correlation was then made to assess whether the difference score was related to the difference in shifting cost between auditory and visual targets, i.e., by subtracting the auditory cost from the visual cost. Pearson’s correlation showed that the difference score in RT between auditory and visual targets was positively correlated with the degree of shifting cost between auditory and visual targets (*r *= .61, *p* = .001).

### Role of stimulus type on error propensity

High false alarms (commission errors) are typical in this paradigm, and are used as a primary measure of performance (Robertson et al., 1997). To interpret whether different types of targets also affected commission errors, a repeated-measures ANOVA compared commission errors between the transition type (shift and non-shift), volume condition (normal and low) and modality of the previous target (auditory and visual). The shift and non-shift occurrences of the 29 visual and 29 auditory NOGO targets were presented randomly and were calculated based on the percentage opportunity for each individual to make an error for that occurrence. The proportion of shift and non-shift trials was similarly distributed by group (*M* = 29, SD = 4) shift trials and (*M *= 29, SD= 4) non-shift trials per person.

The ANOVA showed a significant main effect for transition type *F* (1, 24) = 18.04, *p* < .001, *η*_p_^2^ = .43, indicating more errors occurred following non-shift trials (*M *= 17.8, SD= 15.9%) than shift trials (*M * = 9.2, SD = 9.0%). There was no significant effect for volume condition *F* (1, 24) = 1.61, *p  *= .22, *η*_p_^2^ = .06, or the modality of the previous target *F* (1, 24) = 13.84, *p* = .29, *η*_p_^2^ = .05. There was a significant interaction between transition type and the modality of the previous target *F* (1, 24) = 13.84, *p* = .001, *η*_p_^2^ = .37. For non-shift trials, there was no significant difference between auditory–auditory trials (*M* = 19.6, SD = 15.9%) or visual–visual trials (*M* = 16.1, SD = 15.8%) (Fig. 1c, d). For shift trials, more errors occurred for visual–auditory trials (*M* = 12.9, SD = 10.4%) compared to auditory–visual (*M* = 5.6, SD = 7.6%) (Fig. 4c, d). There were no further significant interactions for transition type and volume (*p* = .89) or for volume and previous target (*p *= .18).

## Discussion

When interpreting the modality-shifting effect, the modality that is being shifted from is suggested to affect the subsequent performance, with greater costs associated with shifting from visual stimuli compared to auditory stimuli (Posner et al., 1976). Here, we show that the dominance effect may be less to do with ‘modality’ per se, and more to do with how long it takes to process the target, relative to the temporal preparation created by the previous stimulus.

Consistent with previous studies, there are shift costs associated with switching between different stimuli (Zubin, 1975; Ferstl et al., 1994; Spence et al., 2001). In the studies that report greater difficulty to shift from visual stimuli, the auditory stimuli were always preceded by a slowly processed visual stimulus (e.g., Posner et al., 1976; Ferstl et al., 1994; Zahn et al., 1994, Sprengelmeyer et al., 1995). In this study, by utilizing stimuli where identification of the auditory stimuli is slower than the visual (inverse of stimuli tested previously), the effect of processing time was explored. The reverse effects are found, in terms of RT, the cost of shifting was greatest to visual targets (processed faster when alone) following auditory ones (processed slower when alone). As an additional control, speed of processing was also manipulated within the auditory modality, with auditory stimuli identified more slowly at low volume intensities. The costs were smallest when the slowest (low volume) stimuli followed a fast visual one, compared to those at the normal intensity. Together, these findings contradict suggestions of a general difficulty in shifting away from visual stimuli (as previously reported), and instead suggest that cost is reduced when targets are preceded by a more rapidly processed stimulus. Individual differences in RT to identifying auditory and visual targets allowed the understanding of processing time to be explored further. Correlation analysis showed that the difference in RT between identifying the auditory and visual targets was significantly related to the difference in cost. The current findings suggest that it is the differing processing requirements between the stimuli that appear to mediate the degree of shifting cost.

How might differences in the processing time of the preceding stimulus affect the response to the subsequent stimulus? Consistent with the conflict-monitoring hypothesis, we propose that the systems that sub-serve cognitive control include an evaluative system, continually appraising current demands and conflicts in processing (Botvinick et al., 2001). For non-shift trials, the frequency and recency of modality build up an internal prediction of when the next target will appear (Epstein & Rock, 1960). The temporal preparation and internal prediction of targets are determined by the time the targets are processed and identified, rather than from the onset (Nickerson, 1973). In this study, the visual targets are detected more quickly than the auditory targets in isolation, and the normal volume auditory targets are detected more quickly than the low volume targets. The more established a required response in a frequent and regular pattern, the more anticipated the response associated with the time needed to identify the stimulus (Neisser, 1976; Dekker, 2006; Chamberlain & Sahakian, 2007). RTs become faster when stimuli are repeated for successive trials (Spence et al., 2001). In fixed-pace tasks for non-shift trials, the criterion created by the building priors is quickly engrained and is indicative of a constantly updating speed accuracy trade-off (Peebles & Bothell, 2004; Helton, 2009). The repetitive consistency of the priors establishes a strong tendency for a motor response to be made unless it can be counteracted in sufficient time. Sensory processing time, i.e., the time needed to fully process the identity of a target, is not equivalent to the time a response is initiated, i.e., RT. In the context of a SART when a rare NOGO stimulus is presented, erroneous habitual responses are often initiated before the timing of a target is fully processed, resulting in an error (Hitchcock & Dember, 1999).

When there is a modality-shift, the engrained prediction for time to respond does not match the new stimulus and remapping a new speed/accuracy criterion must be computed, resulting in a cost in RT. In terms of the Conflict-Monitoring Hypothesis model, the system evaluates the present level of conflict, passes them to the centers responsible for control, and activates an adjustment in the strength of influence on future processing (Botvinick et al., 2001). Consistent with other work, a modality-shift leads to an attentional reset, and interrupts the prediction created from a series of congruent targets (Kreutzfeldt, Stephan, Willmes, & Koch, 2016). In the SART, this shift ‘cost’ is likely to be an advantage by delaying the automaticity of response execution, and allowing more time to fully process a target in time to inhibit a habitual response. In line with this, errors are reduced when there is a shift cost compared to those made following non-shift trials. The reduction in errors when a shift occurs demonstrates that sensory processing time continues and the target is appropriately identified.

**Fig. 5:**
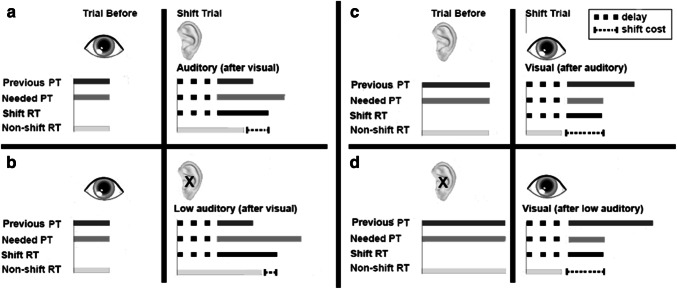
Illustration of ‘underlying predicted’ and ‘needed’ processing time (PT), and ‘shift RT’ in relation to the ‘non-shift RT’ for the cross-modal trials. PT refers to the time needed to correctly identify a target. RT refers to the time people press the response button to the targets. Visuals are exaggerated for illustration purposes only; the ear represents a target that was an auditory digit, and the eye a visual digit. The large dotted line represents the consistent shift, and the fine dotted line represents the changing shift cost. When the previous expectation is faster the ‘shift RT’ is the average of underlying predicted and needed PT. **a** A visual–auditory shift in the normal volume condition. **b** A visual–auditory shift in the low volume condition. **c** An auditory–visual shift in the normal volume condition. **d** An auditory–visual shift in the low volume condition

The size of cost depends on the stimuli that are shifted between. In the current study, when a rapidly processed visual stimulus precedes an auditory stimulus, the switch cost is low. In contrast, when a slowly processed auditory stimulus precedes a visual stimulus, the switch cost is high. Consistent with the temporal preparation account in other paradigms (Los & Van der Burg, 2013), when the underlying predicted processing time of the previous stimulus is faster than the target (in this case auditory after visual/slow after fast), there is a faster response. The left side of Fig. 5a and b shows what happens when the predicted prior processing time allocated to a stimulus is less than that needed to process the stimulus fully. Panel A shows a visual–auditory switch at normal volume. The left side of panel A shows the underlying predicted processing time of the previous visual stimulus based upon the RT of the previous recent target(s) (Epstein & Rock, 1960). The length of the line indicates the time required to identify the visual stimulus from a non-switch trial. The ‘underlying predicted’ and ‘needed’ times are, therefore, shown as the same length of time. The right of panel A shows the switch to an auditory trial from a visual one. The large dotted line indicates a fixed time during which stimulus response reprogramming occurs. For the switch to the auditory trial the ‘underlying predicted’ time is shorter than the ‘needed’ (now shown longer in the second line). The actual shift response time appears to be a compromise between these two times (‘underlying predicted’ and ‘needed’). The final line shows the RT that would occur normally to a non-switch RT, and the capped dashed line shows the difference between this and the shift RT indicating the shift cost. In this context, the observable shift cost is reduced due to a criterion set to respond early. Therefore, the observable effects of the delay created by shifting are effectively shortened by an expectation to respond sooner. A smaller ‘cost’, is more likely to result in an anticipatory response because the response occurs prior to fully identifying the target. Where the shift cost was smaller (visual–auditory) errors were greater, than when the shift cost was largest (auditory–visual).

By comparing to the condition at the reduced volume intensity, the interpretation of these findings as effects of processing time can be further explored (see panel B). Here, we can assess shift costs to targets that are semantically the same but are identified even more slowly. There is now an even slower processed target (low intensity auditory) after the same fast visual target. The longer the processing time needed to identify the shifted target, the more the cost is reduced by the early expectation. Therefore, the observable effects of the delay are effectively shortened even further in Fig. 5b than in Fig. 5a. The further reduction in RT cost observed for volume intensity did not extend to a reduction in error propensity. It is probable that the smaller difference in RT between normal and low volume targets (41 ms), relative to auditory and visual digits (209 ms), was not long enough to create observable benefits in interrupting habitual responses to errors.

Consider now, when the shift occurs the other way (panel C and D), the underlying predicted processing time is much longer than the needed processing time, in this case a fast processed visual stimulus after a slow processed audiotry stimulus. As shown on the right of panel C, the underlying predicted processing time is much longer than the needed processing time. There is now no early expectation to mediate a faster response; instead, the criterion from the RT of the previous target is slower than the target. In this case, the model proposes that following the consistent delay the timing for a speed–accuracy trade-off will always seek the most efficient response. The fast visual target can already be fully identified and a response appropriately initiated in advance of the timing of the slower ‘underlying predicted’ processing time. In other words, after the shift delay occurs, there is no need to set a criterion to ‘wait’ longer for the underlying predicted response time, because the identity of the faster target can be resolved sooner. A slower expectation does not assist in creating an efficient speed–accuracy criterion, and the most efficient response is to fully resolve the identity of the target. The observed shift cost it is now at its maximum, and represents the raw shift delay. By having the second condition with the auditory stimuli at a low intensity, we have the opportunity to assess the impact of having the same fast (visual) target after an even slower (low auditory) target, illustrated in panel D. Here, the post-shift RT to fast (visual targets) after slow (auditory normal volume targets) is comparable to the shift from even slower (low intensity auditory) targets. The shift cost is already at its maximum and is not extended by the expectation of a slower response.

Together, these findings suggest that the expectation effects created by the previous target have a subtractive effect from a shift delay, but only if the underlying predicted response time is faster than the processing time of the target. A speed–accuracy trade-off will always seek the most efficient response time. The longer the processing time needed to identify the shifted target, the more the cost is reduced by an early expectation/fast criterion. Slower preceding targets do not cause an additive effect to the existing cost, because the identity of the subsequent target can be resolved prior to the time of underlying predicted response created by the previous target. Therefore, when the prior prediction is slower, the shift cost represents the raw delay associated with shifting. The findings show how underlying predicted processing time relative to the subsequent target can differentially affect the speed of subsequent responses.

One might interpret that the finding of greater errors to ‘auditory’ stimuli could be explained by greater difficulty to the auditory targets more generally, rather than because they are processed more slowly. However, this does not appear to be the case. In fact, when auditory and visual digits have been used in the same paradigm but in separate tasks, more errors have been observed to the visual targets rather than the auditory (Seli et al., 2012). In this circumstance, the authors suggest that the slower response times may allow for recovery from brief lapses in attention before the next response. Our findings support the assumption that in a task of mixed stimuli greater errors are made to the auditory targets, because they are in the context of stimuli that can be identified faster, rather than something inherent to the stimuli themselves.

To test the hypothesis of the model further, the modality-shifting effect could be explored with stimuli with other parameters. Theoretically, it would be interesting to explore the impact on shift cost if the detection times for the visual and auditory stimuli were comparable. In this context, we would still predict a shift cost for changing modality, but we would not predict differences in shift costs dependent on whether the preceding stimulus was auditory or visual. Such a design to equate processing demand would be difficult to practically achieve between stimuli, and between participants who inherently differ on their speed of responses to stimuli. As such, testing opposing processing times for visual and auditory stimuli, as in the current study, provides a useful way to test if the effect can be reversed, and does not rely on testing a null effect. With this in mind, further comparisons can be made with stimuli that have different stimulus processing demands, or different temporal intervals between stimuli. One consideration could be observing manipulations within a single modality. For example, a mix of normal and low volume targets could manipulate processing time without manipulating semantics or modality. However, based on the current study, the difference in RT between normal and low intensity targets (41 ms) would not be large enough to reliably observe differences in shift RT based on the SDs we see in the current study, and thus we would need a larger processing time manipulation to be able to test response shifts entirely independent of modality.

In light of the current findings, the interpretation of previous studies may also be considered in relation to the processing time due to the selection of stimulus, rather than modality more broadly. As previously discussed, studies which report greater costs to auditory stimuli after shifting from visual have notably used auditory stimuli that can be identified more quickly than the visual stimuli, e.g., (Ferstl et al., 1994; Posner et al., 1976, Zahn et al., 1994, Sprengelmeyer et al., 1995). Consistent with our theory for processing time rather than visual dominance, in a study that instead observed greatest costs when shifting from tactile stimuli (compared to both auditory and visual), the tactile stimuli were identified slowest (Spence, Nicholls, & Driver, 2001). Stimulus-specific sensory processing demands may influence interpretation of findings between tasks and populations. Interpreting shift costs by the speed in which they can be identified is not only potentially important for our interpretation of attentional mechanisms, but also has significant implications for where there are suggestions of sensory dominance/deficiencies, which may be being misinterpreted as modality effects. The findings also have implications for the selection of stimuli in future studies which aim to understand modality processes.

## Conclusion

The key finding in the current work is that responses to stimuli are dependent not only on their own processing requirements, but also on the context of other stimuli within the task. The effects of shifting between stimuli are affected by how long they take to process and how the time of response for the preceding targets affect the following action. ‘Modality-Shifting’ as it is often termed induces shifting costs, but the dominance observed in the costs is not because of a change of modality per se, but because of a change of processing between stimulus types, which mediates a predicted temporal preparation of response. The current findings challenge the assumption that visual dominance in modality-shifting is a consequence of modality, as we can induce greater costs simply by using stimuli with different identification demands. Such a distinction is important to consider when interpreting whether potential deficits may be considered specific to a given modality.

## Abbreviations

SART: Sustained attention to response task
RT: Reaction time
